# Sustained impact of a short small group course with systematic feedback in addition to regular clinical clerkship activities on musculoskeletal examination skills-a controlled study

**DOI:** 10.1186/s12909-016-0554-z

**Published:** 2016-01-28

**Authors:** Martin Perrig, Christoph Berendonk, Anja Rogausch, Christine Beyeler

**Affiliations:** Department of General Internal Medicine, University Hospital of Berne, Berne, Switzerland; Assessment and Evaluation Unit, Institute of Medical Education, University of Berne, 3010 Berne, Switzerland

**Keywords:** Musculoskeletal System, Physical Examination, Clinical Competence, Undergraduate Medical Education, Clinical Clerkship, Feedback, Competency-Based Education

## Abstract

**Background:**

The discrepancy between the extensive impact of musculoskeletal complaints and the common deficiencies in musculoskeletal examination skills lead to increased emphasis on structured teaching and assessment. However, studies of single interventions are scarce and little is known about the time-dependent effect of assisted learning in addition to a standard curriculum. We therefore evaluated the immediate and long-term impact of a small group course on musculoskeletal examination skills.

**Methods:**

All 48 Year 4 medical students of a 6 year curriculum, attending their 8 week clerkship of internal medicine at one University department in Berne, participated in this controlled study. Twenty-seven students were assigned to the intervention of a 6×1 h practical course (4–7 students, interactive hands-on examination of real patients; systematic, detailed feedback to each student by teacher, peers and patients). Twenty-one students took part in the regular clerkship activities only and served as controls. In all students clinical skills (CS, 9 items) were assessed in an Objective Structured Clinical Examination (OSCE) station, including specific musculoskeletal examination skills (MSES, 7 items) and interpersonal skills (IPS, 2 items). Two raters assessed the skills on a 4-point Likert scale at the beginning (T0), the end (T1) and 4–12 months after (T2) the clerkship. Statistical analyses included Friedman test, Wilcoxon rank sum test and Mann-Whitney *U* test.

**Results:**

At T0 there were no significant differences between the intervention and control group. At T1 and T2 the control group showed no significant changes of CS, MSES and IPS compared to T0. In contrast, the intervention group significantly improved CS, MSES and IPS at T1 (*p* < 0.001). This enhancement was sustained for CS and MSES (*p* < 0.05), but not for IPS at T2.

**Conclusions:**

Year 4 medical students were incapable of improving their musculoskeletal examination skills during regular clinical clerkship activities. However, an additional small group, interactive clinical skills course with feedback from various sources, improved these essential examination skills immediately after the teaching and several months later. We conclude that supplementary specific teaching activities are needed. Even a single, short-lasting targeted module can have a long lasting effect and is worth the additional effort.

## Background

Musculoskeletal complaints are highly prevalent with great impact on quality of life and socioeconomic costs due to outpatient and inpatient care, inability to work and disability [1]. Clinical skills are vital for physicians to provide good quality patient care [2]. It has been repeatedly demonstrated that an accurate history and physical examination lead to the exact diagnosis in more than 80 % of all cases [3–5]. However, novice physicians may be incompetent in these basic clinical skills [6]. These shortcomings seem to be particularly apparent in musculoskeletal medicine [6–8] and result in missing locomotor symptoms and signs, and omitting of treatment of symptomatic patients [9, 10].

Several studies concluded that “medical school preparation in musculoskeletal medicine is inadequate” [11–14]. Consequently, several calls for the development of core recommendations for undergraduate medical education curricula of the minimum level of competence in managing patients with musculoskeletal problems were expressed [11, 15, 16]. These appeals lead to increased emphasis on structured teaching and assessment of musculoskeletal skills [17, 18]. The GALS (Gait Arms Legs and Spine) test [19] is such an example of a well-structured, quick screening examination of the musculoskeletal system and is a sensitive and valid indicator of musculoskeletal functional ability [20]. It has been shown that by incorporating GALS in the undergraduate medical curriculum, Year 3 medical students performed well in an end of year musculoskeletal station of an Objective Structured Clinical Examination (OSCE) [21]. However, this effect’s generalization was questioned [22] and a call for implementing a standardized approach to musculoskeletal clinical teaching superseding GALS was suggested [23].

Once an abnormality of the musculoskeletal system has been identified by a screening functional test, a more detailed regional examination has to be performed [24]. However, it is less clear which particular skills have to be mastered [25, 26]. For that very reason, core sets of regional musculoskeletal examination skills were put together for medical students at the point of qualification [26, 27]. In addition, communication skills and professional attitudes were incorporated into the recommended basic competencies [16].

Nevertheless, the question of how these core competencies in musculoskeletal examination skills can be taught and assessed in the demanding clinical setting remains a matter of ongoing search. A best evidence in medical education systematic review of strategies and their effectiveness summarized published studies on structured educational interventions in undergraduate and postgraduate settings [28]. There was support that small-group instructions with supervision and feedback were core elements for an effective teaching of clinical skills in musculoskeletal medicine [29, 30]. Several studies have investigated practical interactive learning with patients, tutors and peers within curricular series with mixed teaching formats [31–37]. However, results of single interventions are scarce. Furthermore, little is known about the time-dependent effect of assisted learning in addition to a standard curriculum. To our knowledge only few studies documented a favorable effect of a supplementary approach to musculoskeletal system training at the time of an end-of-year OSCE [21, 38] or even later on [29].

We therefore addressed the following questions in this study:

1. To what extent do medical students’ musculoskeletal examination skills improve during regular undergraduate clerkships assessed by structured observation?
2. What is the impact of an additional 6-h interactive course (embedded in the regular clerkship activities) on musculoskeletal examination skills?
3. In case of an added impact, does the success persist over several months after the intervention?

## Methods

### Regular teaching and learning activities

This controlled intervention study was carried out at the University of Berne, Switzerland, where a 6 year undergraduate medical training program takes place. Years 1–3 are built up of comprehensive lectures, problem based tutorials and specific clinical skills training. During this preclinical period, musculoskeletal examination is taught in a 4×2 h skills training in Year 3. This is a small group teaching with written learning objectives, various learning materials and hands-on exercises among peers without involving patients, and with feedback by teachers and peers. At the end of Year 3 these skills are assessed by a specific 10 min station within an interdisciplinary Objective Structured Clinical Examination (OSCE) test.

Years 4–6 consist of lectures and clinical training in various forms and settings. There are two clerkship periods. The first one situated in Years 4 and 5 included 9 different clerkships at the time of the study in 2006 and 2007: internal medicine (8 weeks), surgery (6 weeks), pediatrics (6 weeks), pathology (6 weeks), gynecology and obstetrics (4 weeks), psychiatry (4 weeks), dermatology (3 weeks), ENT (3 weeks) and ophthalmology (3 weeks). The students rotate in small groups (up to 7 individuals, each group in a different order and in different teaching hospitals). The second one is the elective period in Year 6, where students can choose different clerkships during a total of 40 weeks.

Our study was situated in the 8-week internal medicine rotation of the first clerkship period in one teaching hospital. During this period the students are supposed to gain experience in history taking, physical examination, inter-professional and patient communication as well as professional behavior. The students are aware of the Swiss Catalogue of Learning Objectives for Undergraduate Medical Training (SCLO) [39] and have access to various learning materials such as books, scripts, e-learning programs and the interdisciplinary skills centre. During this clerkship the students collaborate with the medical team in the everyday clinical work. Typically, there is one-to-one, formally unstructured teaching by the assigned resident, yet without systematic supervision and without regular feedback as a matter of routine.

### Subjects

The subjects of this study consisted of Year 4 medical students of the University of Berne. Each year the students’ office allocated four clerkship groups composed by convenience to the Department of General Internal Medicine, University Hospital of Berne, with a total of 26 students out of 154 in 2006 and 22 students out of 153 in 2007. This procedure was completely independent of this study. Each year, two out of the four clerkship groups were allocated to the intervention described below. This block allocation was dependent on the availability of the teacher only. She was neither given any information about the participants nor had any influence on their allocation. Thereby, in 2006 the second and third clerkship group (March–June), and in 2007 the first and second clerkship group (January–April) took part in the intervention, resulting in a total of 27 students. The other four clerkship groups served as controls with a total of 21 students. At the beginning of the clerkship all 48 students received the learning objectives and references of various learning materials specific for the musculoskeletal examination. The 21 control students were not offered supplemental faculty attention in addition to the regular teaching activities described above. The study protocol and all additional documents requested - including assessment forms, information sheets and consent forms - were presented to the responsible ethical committee of the Medical Faculty of the University of Berne. The “Kantonale Ethikkommission” KEK [40] decided that the study was exempt from formal ethical approval because the relevant legislations related to drugs and clinical research were not concerned. All students volunteered to participate and gave written informed consent.

### Intervention

The intervention consisted of an interactive course about the examination of the musculoskeletal system integrated into the regular clerkship activities described above. The students were taught in small groups with 4–7 participants during 6 lessons of 1 h each based on the identical learning objectives and learning materials applied to the regular teaching in Year 3. The teacher was an experienced specialist in rheumatology. For every lesson an inpatient from the Department of Internal Medicine volunteered to participate. Each lesson focused on the examination of one or two regions of the musculoskeletal system: shoulder, elbow, hand, hip, knee, foot and back. The essential and important aspects of the physical examination were covered including the correct verbalization of the findings. Special emphasis was also put on the interaction with the patient, i.e., how to communicate with the patient and to behave in a professional manner. Each student was expected to actively practice the skills either with the patient or with a peer, followed by formative feedback from the patient, the peers and the tutor (“supported participation” [41]).

### Assessments

The students’ competencies in musculoskeletal examination were assessed at the beginning (T0), the end (T1) and 4–12 months after completion of this particular clerkship (T2) by an OSCE-like 10 min testing station [42, 43]. The students were asked to perform different tasks with a standardized patient and to summarize their findings. The students were familiar with this assessment format after passing the Year 3 OSCE one year before. However, no practice runs were offered - neither to the intervention nor to the control students. The tasks were similar but not identical to the tasks which had been covered during the instructional course. Therefore, the tasks were deliberately chosen in order to avoid the teaching to the test phenomenon. The students were rated with respect to 9 clinical skills (CS; Table 1). Seven skills were related to specific aspects of the musculoskeletal examination (MSES): inspection, palpation, joint and spine movements including documentation, trigger tests and verbalization of physical findings. Two skills were related to interpersonal skills (IPS): communication with the patient and professional behavior. The competencies were assessed on a 4-point Likert scale based on specified criteria by two independent raters. In 2006, for each assessment session the team of two raters was recruited out of five different specialists, three of whom were not blinded to the intervention due to logistic reasons. In 2007, the raters were two specialists in general internal medicine blinded to the intervention. All were experienced clinicians and had been teaching and examining for several years. The teacher of the musculoskeletal examination course was deliberately not acting as a rater.

**Table 1:** Inter-rater agreement within the two study cohorts (2006 and 2007)

Task		Kendall’s Tau-b (2006)			Kendall’s Tau-b 2007)		
		T0	T1	T2	T0	T1	T2
Clinical Skills (CS)	Musculoskeletal Examination Skills (MSES)						
	Inspection	0.79	0.57	0.78	0.59	0.53	0.64
	Palpation of anatomic landmarks	0.46	0.81	1.00	0.52	0.49	1.00
	Movements of one joint	0.81	0.69	(.)^a^	0.81	0.80	(.)^a^
	Documentation of joint movements	0.67	1.00	0.78	0.84	0.83	0.58
	Movements of the spine	0.78	0.76	0.98	0.77	0.64	0.54
	Trigger test	0.71	0.72	0.67	0.59	0.70	0.80
	Verbalization of physical findings	0.54	0.47	0.63	0.50	0.55	0.75
	Interpersonal Skills (IPS)						
	Communication with patient	0.63	0.53	0.65	0.31	0.74	0.30
	Professional behavior	0.41	−0.22	0.27	0.20	0.68	0.57

^a^ Not computable due to non-varying ratings of one rater

### Statistical analyses

Internal consistency (Cronbach’s Alpha) and inter-rater agreement (Kendall’s tau-b) were examined within the two study cohorts separately. Subsequently, ratings of the two raters were averaged for further statistical analyses. Means of all 9 ratings were computed to a total score of clinical skills (CS). In addition, the 7 ratings related to the musculoskeletal examination skills (MSES) and the 2 ratings related to the interpersonal skills (IPS) were calculated separately (means and standard deviations are presented). As students of the cohorts 2006 and 2007 showed comparable improvements from T0 to T1 and T2, it was justified to jointly analyze the two study cohorts as a combined sample.

Statistical analyses included Friedman tests for the differences between the different measuring times (T0, T1, T2) within both groups (control and intervention), followed by Wilcoxon rank sum tests for more detailed analyses. To analyze differences between the intervention and control group as well as a possible bias in dropouts, students’ performance was compared between these groups by Mann-Whitney *U* test. Statistical analyses were performed using SPSS, Version 15. The level of significance was set at *p* = 0.05 with two sided testing.

## Results

### Sample characteristics

All 48 students (52 % female; mean age 25 years) attending their clerkship in internal medicine at the Department of General Internal Medicine, University Hospital of Berne in 2006 and 2007 were enrolled in this study: 27 students were allocated to the intervention group and 21 students to the control group.

**Fig. 1:**
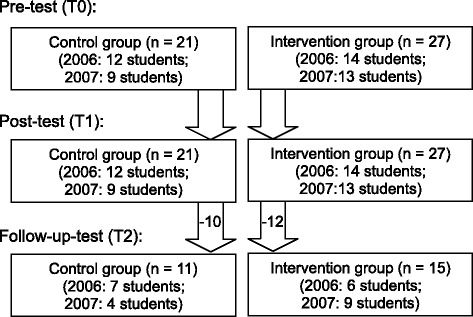
Sample of the study participants in 2006 and 2007

All students participated in the pre-test (T0) at the beginning and in the post-test (T1) at the end of the 8-week clerkship. On average, the control students had attended a slightly longer period of clinical training before T0 compared to intervention students (5.5 and 3.5 months in clerkship, respectively). The control and intervention students were comparable regarding attending in clerkship specialties related to the musculoskeletal system prior to participating in the study. About three quarters of the students (*n* = 35, 73 %) had already participated in a clerkship in surgery, orthopedics or rheumatology before the baseline assessment T0. About half of the students (*n* = 26, 54 %) took part at the follow-up-test (T2), (see Fig. 1), which on average took place 6.5 months (control group) and 8.5 months (intervention group) after T1 respectively. No significant differences in pre- or post-test performances (T0, T1) were found between students participating in the follow-up-test (T2) and those who dropped out.

### Inter-rater agreement and internal consistency

For the assessments in 2006, the Cronbach’s Alpha for the 9-item test at T0 was 0.61, at T1 0.47 and at T2 0.78, respectively. The corresponding values for 2007 were 0.73 (T0), 0.80 (T1) and 0.83 (T2), respectively. Table 1 shows the inter-rater reliability for each test item at the different testing times within the two study cohorts (2006 and 2007).

### Change of skills over time

At the beginning of the clerkship (T0), the intervention group (CS: 2.97 ± .40; MSES: 2.85 ± .46; IPS: 3.38 ± .50) and the control group (CS: 3.12 ± .44; MSES: 3.05 ± .48; IPS: 3.35 ± .44) performed equally (Mann-Whitney *U* test: n.s.). The intervention group changed markedly in its performance over time (Friedman test *p* < 0.001 for CS, *p* < 0.001 for MSES and *p* < 0.01 for IPS), whereas the control group did not (Friedman test for CS, MSES and IPS: all n.s.).

### Effect of the intervention

**Fig. 2:**
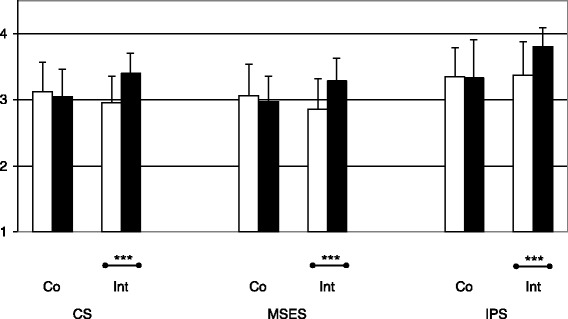
Effect of the interactive small group course immediately after the clerkship. Mean and standard deviation of clinical skills (CS), musculoskeletal examination skills (MSES) and interpersonal skills (IPS) of 48 students within the control (Co) or the intervention (Int) group before (T0, □) and immediately after (T1, ■) the clerkship. *** *p* < 0.001

In detail, the intervention group showed significantly higher skills compared to baseline T0 with respect to all criteria after participation in the interactive course (T1 CS: 3.40 ± .30, Wilcoxon test *p* < 0.001; MSES: 3.29 ± .33, *p* < 0.001; IPS: 3.80 ± .29, *p* < 0.001), (Fig. 2). In contrast, the control group did not show a change in these specific skills during the clerkship (T1 CS: 3.05 ± .41; MSES: 2.97 ± .39; IPS: 3.33 ± .58, all n.s.). Thus, at T1 the two groups differed with respect to all dimensions (Mann-Whitney *U* test: CS: *p* < 0.01; MSES: *p* < 0.01; IPS: *p* < 0.01). Analyzing the two cohorts of 2006 and 2007 separately did not change the results, but showed the same effect of the intervention.

**Fig. 3:**
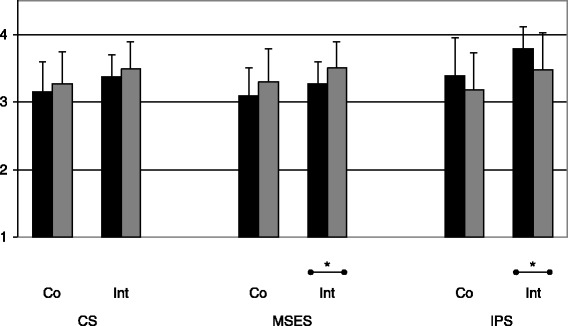
Effect of the interactive small group course several months after the clerkship. Mean and standard deviation of clinical skills (CS), musculoskeletal examination skills (MSES) and interpersonal skills (IPS) within the control (Co) or intervention (Int) group of those 26 students who completed the assessment immediately after the clerkship (T1, ■) and several months later (T2, gray box). * *p* < 0.05

On follow-up-test (T2), the intervention group still showed an enhanced proficiency compared to baseline (T0) for clinical skills (CS: 3.49 ± .40; Wilcoxon test *p* = 0.001) and musculoskeletal examination skills (MSES: 3.50 ± .39; *p* = 0.001), but not for interpersonal skills (IPS: 3.47 ± .55), which deteriorated during follow-up. The differences between the post-test (T1) and the follow-up-test (T2) were in part significant (CS: *p* < 0.10; MSES: *p* < 0.05; IPS: *p* < 0.05). In contrast, the control group did not show any significant improvement over time (T2 CS: 3.27 ± .48; MSES: 3.30 ± .49; IPS: 3.18 ± .55), but remained on the same skills level (Fig. 3).

## Discussion

This study assessed to what extent medical students’ musculoskeletal examination skills improved during regular undergraduate clerkship in internal medicine and explored the impact of an additional 6-h interactive small group course embedded in the everyday clerkship activities.

At the beginning of the clerkship all students demonstrated basic musculoskeletal examination skills as a result of the clinical skills training during Year 3. Although, on average, the students in the control group had attended a slightly longer period of clinical training before the first assessment applying structured observation (T0), they did not show better baseline performance compared to the students in the intervention group. In addition, there was no significant increase in these clinical skills detectable, neither immediately after the clerkship in internal medicine nor at the end of the whole clerkship period with rotations through various disciplines over a total of 43 weeks. In contrast, the students attending an additional 6-h course integrated into the clerkship in internal medicine showed significantly improved musculoskeletal examination skills. The benefit of this specific intervention was detectable both immediately and several months after the course.

Our findings illustrate that Year 4 medical students are not able to further improve their musculoskeletal examination skills by their own prompting and indicate the limited influence of regular clerkship activities. Bloomfield et al. analyzed the learning activities of final year students and concluded that clinical clerkships would become more efficient when goals, teaching/learning methods and assessments are brought into better alignment as the students’ learning activities seemed to be driven by the assessments [44]. Nevertheless, our control students showed no improvement of their clinical skills although they were explicitly referred to the learning objectives and various learning materials specific for the musculoskeletal examination skills, even though they knew about the test at the end of the clerkship. This assessment obviously had no significant effect on their acquisition of musculoskeletal examination skills. The kind of assessment might play an important role, since our tests were purely formative in contrast to the summative final examinations mentioned in Bloomfield’s study.

The ineffectiveness of the routine clerkship in respect to the acquisition of musculoskeletal examination skills might also be explained by the observation that many regular clinical teaching activities are passive, low-level cognitive actions and do not provide an ideal learning environment [45–47]. In addition, students might not be aware of their gaps in learning, since confidence does not necessarily reflect competence [48]. However, students appreciate active learning including coaching, feedback (in particular feedback on history taking and physical examination [49]) and supervision while interacting with patients [50]. In addition, there is some evidence that the time spent on activities involving direct patient contact is positively related to students’ perceptions of the quality of their learning environment [51]. In order to reduce the gap between what is most valued and what is delivered in the clinical context protected teaching time with close student-teacher interaction is warranted [47]. Medical schools cannot rely on clerkship experiences alone to achieve basic skills training but need to offer additional support [52]. Duvivier et al. findings on the influence of the workplace on learning physical examination skills can direct future developments [53]. Their qualitative approach with focus groups identified helpful factors such as making findings explicit through patient files or during observation, feedback by abnormal findings and taking initiative. Hindering factors included lack of supervision, uncertainty about tasks and expectations, and social context such as hierarchy of learners and perceived learning environment.

We introduced an interactive course. The core elements were small-group teaching by a specialist, hands-on examination of real patients, and in particular supervision with systematic and detailed feedback by teacher, peers and patients. We confirmed that these are important elements indeed to improve musculoskeletal examination skills in line with Dolmans’ et al. results according to which high-quality supervision improved the effectiveness of clinical rotations [30]. Our findings also correspond to the results of Lawry et al. who demonstrated that small-group teaching was superior to other methods to train Year 2 students in musculoskeletal screening skills, in particular regarding the persistence of the skills over several months [29]. Our data confirm that specific small group teaching in combination with other elements have a long lasting effect on the improvement of musculoskeletal examination skills of Year 4 students. However, our findings are not consistent with the results of Smith et al. who found structured clinical instruction modules to be partly inferior to small bedside group tutorials [54]. Although, those groups were not comparable in respect to teaching time (3 versus 20 h) and group size (24–26 versus 9–10 students), factors essential for the teaching effectiveness.

We chose a specialist in rheumatology as teacher for our course. She was familiar with the curriculum and the expected level of competence of Year 4 students. Due to limited teaching resources some authors involved other individuals in the teaching of musculoskeletal examination skills to medical students. They could demonstrate that patient partners [55], patient educators [56–61], nurses [62] and physiotherapists [63] were as effective teachers as physicians, or neither better or worse, but complementary to physicians [64, 65]. Even the participation of medical students as teachers in a structured peer-assisted learning is another option to enhance clinical examination skills training [66, 67]. Whether non-academic teachers could also achieve the long-lasting effect of a short 6-h intervention on musculoskeletal examination skills over several months as demonstrated by this paper, needs to be studied further. In addition, comparison with the introduction of workplace based assessments like the Mini-Clinical Evaluation Exercises (Mini-CEX), characterized by observations during regular clinical practice followed by feedback [68], could analyze the effect on musculoskeletal examination skills. In any case, effective measures have to be taken at counteracting the decline of physical examination skills observed over the last decades [69] and the resistance to change in daily practice [70].

There are some limitations to our study. First, the students’ scores at the beginning of the clerkship were already at the upper range of the rating scale suggesting that a further improvement would be difficult to demonstrate (ceiling effect). Nevertheless, the results of the OSCE showed a significant increase from T0 to T1 in the intervention group compared to the control group even with respect to the interpersonal skills that were rated with the highest scores. Second, for more advanced students with increased clinical competence, global scores might be more appropriate compared to detailed checklists [71]. Third, we found an immediate benefit of the course on communication skills and professional behavior, which declined after several months. This finding has to be interpreted with some caution. The items regarding communication and professional behavior were rather unspecific and left room for interpretation. In addition, the inter-rater agreement was quite low. For future studies, more specific rater training regarding the assessment of communication and professional behavior or the use of a more specific instrument might be useful. Fourth, in 2006 three out of five raters were not blinded to the intervention due to logistic reasons. Nevertheless, the improvement of the OCSE scores over time from T0 to T1 and T2 were comparable in 2007, where the two raters involved were blinded to the intervention. Fifth, whereas all students took part in the T0 and T1 assessment at the beginning and the end of the clerkship, 54 % only participated in the completely optional follow up assessment T2 several months later. Although at this time the students were present on the campus for lectures, competition with preparing summative exams, earning money and various other activities became evident. Nevertheless, the dropout observed was independent of pre- and post-test performance.

## Conclusions

In conclusion, Year 4 medical students had difficulties improving their musculoskeletal examination skills during their regular clerkship activities. However, an additional short interactive small group teaching course with hands-on examination of real patients and feedback from various sources was effective to the learning of these essential skills with a sustained impact over several months.

## Abbreviations

CS: clinical skills
GALS: gait arms legs and spine test
IPS: interpersonal skills
MSES: musculoskeletal examination skills
OSCE: objective structured clinical examination
T0: time at the beginning of the clerkship
T1: time at the end of the clerkship
T2: time several months after the clerkship
